# The role of intraoperative hyperspectral imaging (HSI) in colon interposition after esophagectomy

**DOI:** 10.1186/s12893-023-01946-3

**Published:** 2023-03-02

**Authors:** Anne Zimmermann, Hannes Köhler, Claire Chalopin, Boris Jansen-Winkeln, Robert Nowotny, Till Schönherr, Matthias Mehdorn, Konstantin Lukas Uttinger, René Thieme, Ines Gockel, Yusef Moulla

**Affiliations:** 1 Department of Visceral, Transplant, Thoracic and Vascular Surgery, University Hospital Leipzig, Liebigstr. 20, D-04103 Leipzig, Germany; 2 Innovation Center Computer Assisted Surgery (ICCAS), University of Leipzig, Semmelweisstr. 14, D-04103 Leipzig, Germany; 3 Department of General, Visceral, Thoracic and Vascular Surgery, St. Georg Hospital, Delitzscher Str. 141, D-04129 Leipzig, Germany

**Keywords:** Colon interposition, HSI, Anastomotic leak

## Abstract

**Background:**

Colon conduit is an alternative approach to reconstructing the alimentary tract after esophagectomy. Hyperspectral imaging (HSI) has been demonstrated to be effective for evaluating the perfusion of gastric conduits, but not colon conduits. This is the first study to describe this new tool addressing image-guided surgery and supporting esophageal surgeons to select the optimal colon segment for the conduit and anastomotic site intraoperatively.

**Patients and methods:**

Of 10 patients, eight who underwent reconstruction with a long-segment colon conduit after esophagectomy between 01/05/2018 and 01/04/2022 were included in this study. HSI was recorded at the root and tip of the colon conduit after clamping the middle colic vessels, allowing us to evaluate the perfusion and appropriate part of the colon segment.

**Results:**

Anastomotic leak (AL) was detected in only one (12.5%) of all the enrolled patients (n = 8). None of the patients developed conduit necrosis. Only one patient required re-anastomosis on postoperative day 4. No patient needed conduit removal, esophageal diversion, or stent placement. There was a change in the anastomosis site to proximal in two patients intraoperatively. There was no need to change the side of colon conduit intraoperatively in any patient.

**Conclusion:**

HSI is a promising and novel intraoperative imaging tool to objectively assess the perfusion of the colon conduit. It helps the surgeon to define the best perfused anastomosis site and the side of colon conduit in this type of operation.

## Introduction

The current gold standard for esophageal replacement after resection is to employ a gastric conduit. However, an alternative interposition, such as a colon conduit, is sometimes necessary when the stomach cannot be used, such as post-gastrectomy, simultaneous gastric carcinoma, in cases with a disturbed or altered gastro-epiploic arcade, or previous upper abdominal surgery [1–3]. A major success factor when using a colon conduit as an esophageal substitute is an adequate blood supply. The blood supply of the left colon relies on the marginal artery of Drummond (branch of the inferior mesenteric artery [IMA]). In contrast, a right colon conduit depends on the middle colic branch of the superior mesenteric artery (SMA) [4, 5]. The challenge is evaluating and preserving the colon conduit’s arterial inflow and venous drainage intraoperatively. Any minor misjudgment or technical mistake can lead to disastrous complications. Anastomotic leak (AL) and colon necrosis are major postoperative complications [6, 7]. AL remains the main cause of increased morbidity and mortality in esophageal surgery [7]. A sufficient blood supply to the colon conduit is essential for anastomotic healing and reduces the postoperative AL rate. Different methods have been described to evaluate the colon conduit’s perfusion intraoperatively. The anastomosis can usually be assessed macroscopically via indocyanine green fluorescence angiography (ICG-FA) or Doppler ultrasound (US) [8–12].

However, none of these examination methods has been shown to analyze colon perfusion sufficiently when employed interpositionally after esophagectomy.

Hyperspectral imaging (HSI) is a non-invasive, contact-free, non-ionizing tool used to measure indicators of tissue perfusion. The HSI system delivers colored images of physiological tissue parameters, such as oxygen saturation (StO_2_) and tissue perfusion (Near-Infrared Perfusion Index [NIR-PI]), which can guide the surgeon intraoperatively and impact the surgical outcome [13, 14]. In this study, we aimed to evaluate the benefit of using HSI in predicting colon conduit perfusion when it is used as a substitute after esophageal resection.

## Methods

### Study design

This single-center, one-arm, prospective observational study was designed in accordance with the Declaration of Helsinki. The study was approved by the local ethics committee at the Medical Faculty of the University of Leipzig (026/18-ek) and registered at Clinicaltrials.gov (accessed on February 22, 2020) (NCT04230603).

.

### Inclusion criteria

All adult patients who underwent long-segment colon interposition with cervical anastomosis following esophagectomy between 01/05/2018 and 01/04/2022 were included. Patients without HSI measurements were excluded. Included patients were informed about the procedures in detail and provided their written informed consent.

### Hyperspectral imaging

HSI data were acquired via the TIVITA® Tissue System (Diaspective Vision GmbH, Am Salzhaff, Germany) under standard conditions [15]. Operation time was not significantly influenced by the application, as the data collection only takes about 10 s.

In brief, the HSI camera is positioned at a fixed distance 50 cm above the region of interest (ROI), and all ambient lights were switched off, keeping artifacts to a minimum. The object was illuminated by six integrated halogen spotlights, allowing spectral data acquisition in the visible and near-infrared spectral range from 500 to 1000 nm with a push-broom HSI camera. The integrated analysis software calculates one RGB color image and four false-color images depicting perfusion parameters (NIR-PI and StO_2_), tissue water index (TWI), and organ hemoglobin index (OHI) with an effective number of 640 × 480 pixels. The field of view (FOV) and spatial resolution depend on the objective lens and distance used. With this setup, a 8 × 6.5 cm^2^ FOV and theoretical spatial resolution of 0.13 mm/pixel are achieved [13, 16].

### Surgical technique

The reconstruction with colon conduit was performed after esophagogastrectomy during the same surgical procedure as a one-step procedure or at a different time with an interval, as a two-step procedure. Left-sided colon interposition was intended in all included patients. The preparation of colon interposition is described in the following text.

When considering the colon as a potential esophageal substitute, colonoscopy is performed routinely in our clinic to assess the colon mucosa and to disclose any pathologies. Furthermore, angiography or computed tomography angiography (CTA) is performed to evaluate the abdominal vasculature, especially the SMA and IMA and their branches to the colon. We usually use the left-sided long-segment colon with cervical anastomosis for reconstruction, as described by DeMeester et al. [1],

where its blood supply depends mainly on adequate inflow from the IMA. No patient in our cohort underwent right-sided colon interposition. The procedure starts with a median laparotomy in the upper and middle abdomen. The avascular peritoneal fold (Toldt line) and splenocolic ligament are then dissected, and the left colonic flexure is mobilized away from the retroperitoneum. The greater omentum is then separated from the transverse colon. The right colon is then mobilized similarly. After the colon frame has been completely mobilized, the middle colic artery is identified and test-clamped close to the root.

The distal transverse colon is then pulled up to the xiphoid and marked at this point with a suture (this represents the aboral anastomosis). The distance between the marked suture point, the planned area of the cervical anastomosis, is then measured and drawn up to the ascending colon and marked with a second suture, which is usually at the mid-ascending colon. At this point during the surgery, we check the conduit’s perfusion macroscopically and by using HSI to obtain objective information on the perfusion situation at both marked points. Once the left colon’s perfusion is deemed adequate, the colon mesentery is skeletonized step by step, and the middle colic vessels are ligated and divided close to the root.

**Fig. 1:**
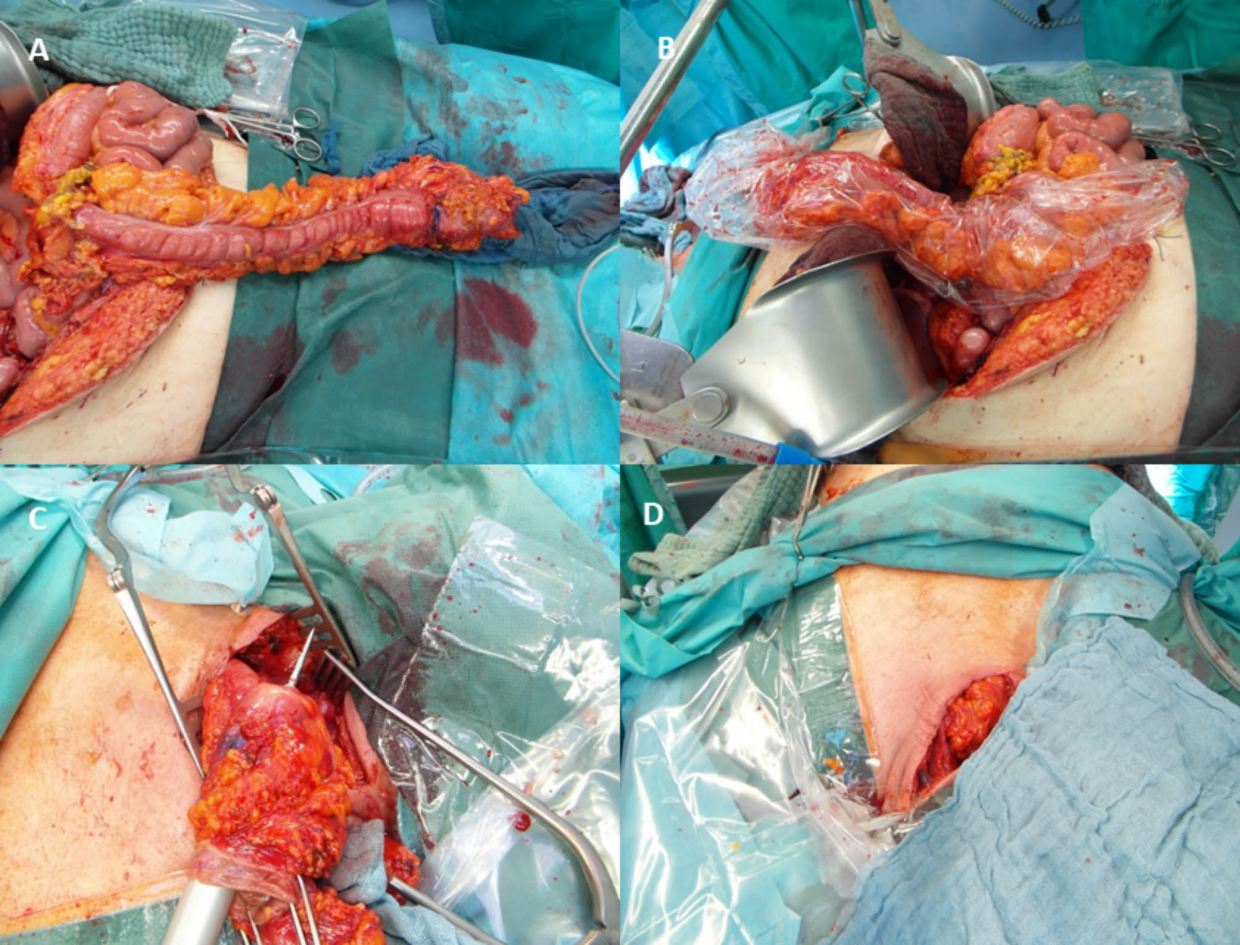
Surgical steps of colon interposition left side; *A + B* Mobilization and covering of colon conduit; *C + D* cervical stapled- anastomosis

The colon is first set down at the oral marker using a stapler. This end is then knotted with a long suture, which is then passed retrosternally to the neck area, and after being covering with a plastic bag for protection, the colon is slowly pulled into the neck area via the retrosternal route without any tension (Fig. 1). The perfusion parameters (NIR-PI and StO_2_) are checked again at the base and the tip of colon conduit to reveal any disruption of the colic arcade after pulling up the colon conduit.

### Cervical phase

The upper limit of the anastomosis site is prepared and marked with a blue line (skin marker, Symmetry Surgical GmbH, Germany) or with a forceps at the end of the colonic arcade (Fig. 6). The anastomosis site below the marked line is evaluated subjectively and via HSI measurements. The site of the anastomosis is adapted according to the HSI measurements in the case of insufficient perfusion of the colon tip according to the subjective evaluation. Then, the half of the manubrium and the left clavicle’s sternal head are removed, as described by DeMeester et al. [1].

An esophago-colonic anastomosis can be made via many techniques; if possible, we prefer a stapler anastomosis with EEA 25 mm in our department. In some cases, this was also performed via an end-to-end hand-sewn technique using a single layer of interrupted 4 − 0 monofilament sutures.

### Abdominal anastomoses

To reconstruct the alimentary tract, we make either an end-to-side manual anastomosis between the colonic conduit and residual stomach or a jejunal Roux-en-Y anastomosis (colon-jejunum). The colon’s continuity is ultimately restored with an end-to-end manual anastomosis using continuous 4 − 0 monofilament sutures or with a side-to-side stapled anastomosis.

### Intraoperative hyperspectral imaging (HSI)

**Fig. 2:**
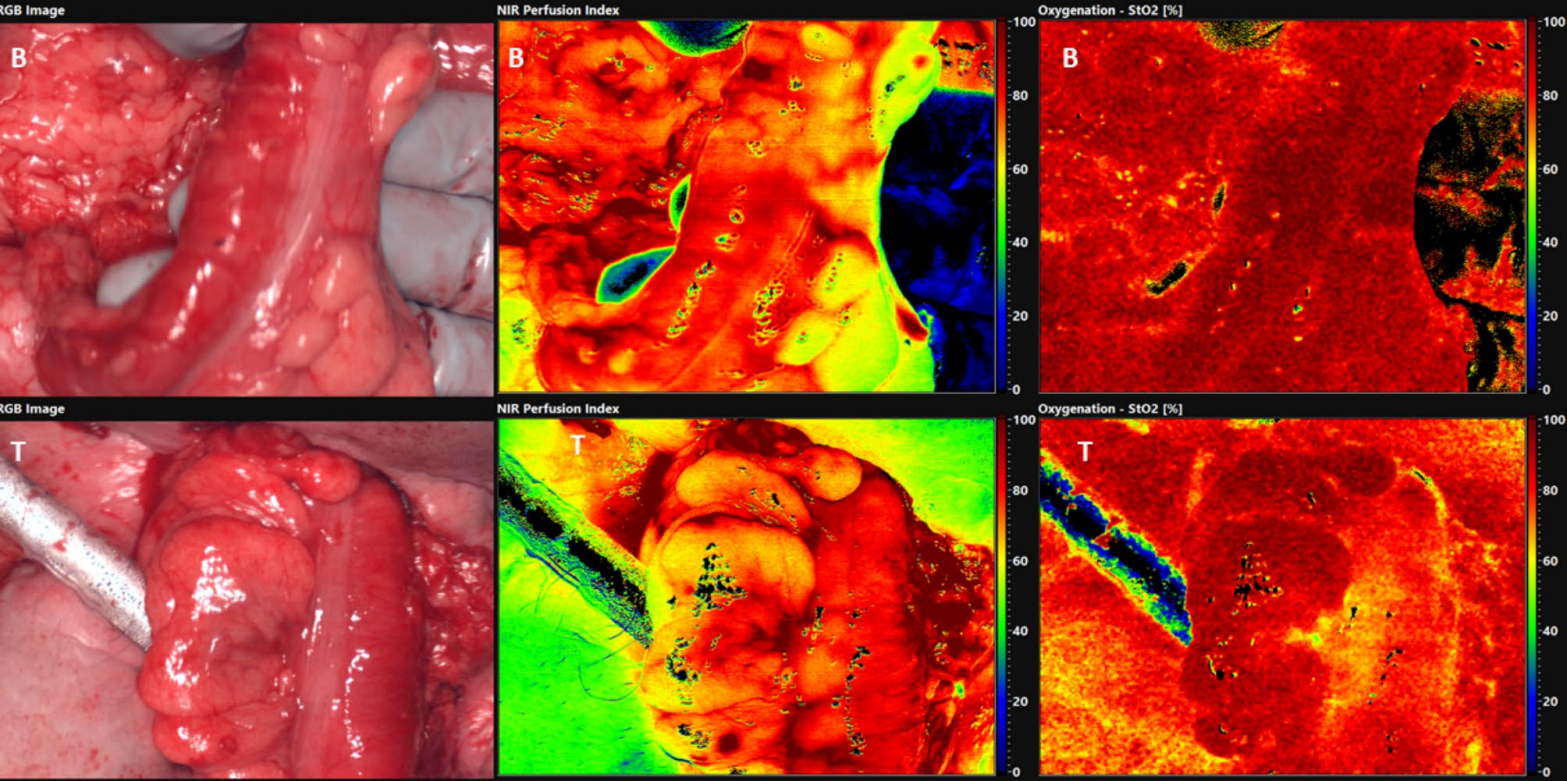
Intraoperative HSI-Measurements, *B* base of the colon interposition, *T* tip of the colon interposition in cervical area, *left;* the colored images, *middle;* color-coded images of NIR-PI, *right;* color-coded images of StO2

**Fig. 3:**
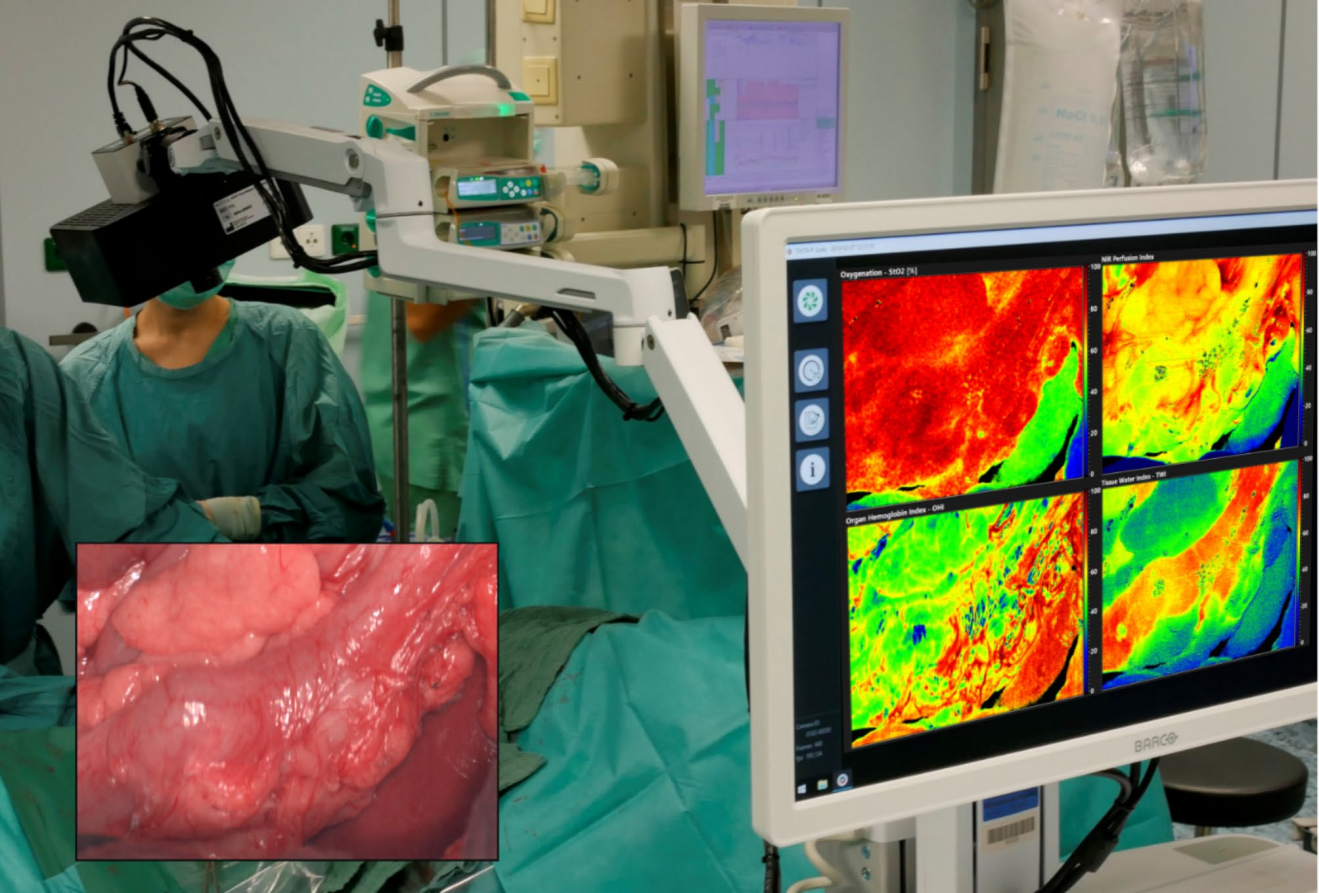
TIVITA® device and HSI camera in the operative setting. *Right*; The chemical color imaging procedure is calculated with the attached computer. *Bottom-left*; operative site

HSI is a routine procedure for evaluating the perfusion of abdominal organs and the intestine in our clinic. For this purpose, appropriate false-colored images of tissue parameters are obtained from HSI before and after forming the colon conduit. The lights in the operating room are switched off for approximately 10 s. Subsequently, larger overview HSI images of the colon conduit display the perfusion and oxygenation after clamping of the middle colic artery (e.g., false-colored images show a red-orange color of the conduit as a sign of good perfusion, yellow color as a sign of moderate perfusion, and blue-green color as a sign of poor perfusion). Moreover, HSI images of the base and tip of the colon conduit are taken to detect the perfusion after pulling up the colon conduit (Fig. 2). Furthermore, the HSI data are analyzed using the TIVITA® Suite software to calculate the means of the tissue parameters inside the ROI (Figs. 2 and 3).

### Clinical pathway

**Fig. 4:**
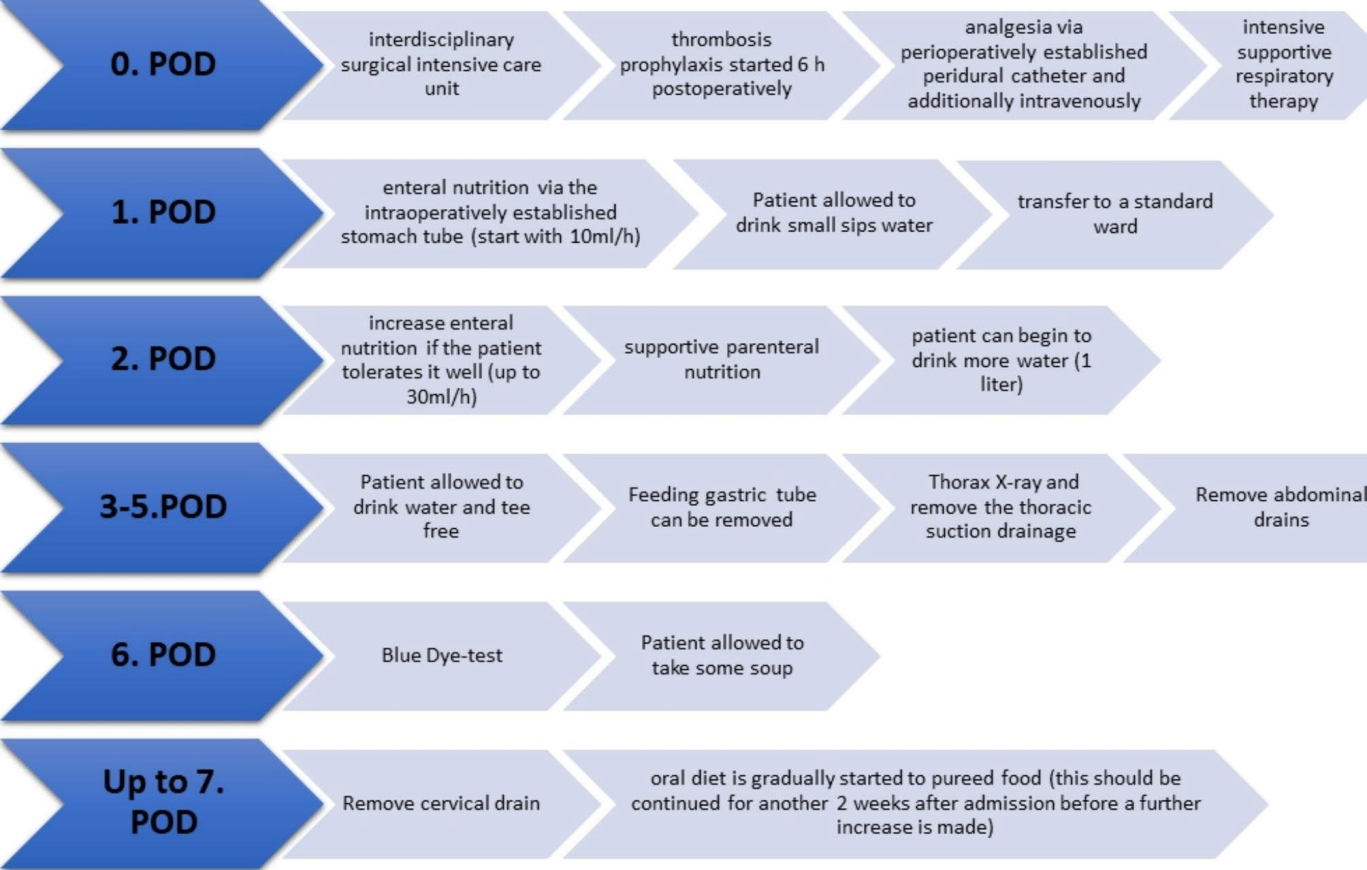
Postoperative treatment in our Department according to our standardized pathway. The figure shows the individual steps according to postoperative days (POD)

All our patients were treated according to our standard perioperative clinical pathway (Fig. 4).

### Follow-Up and endpoints

We collected follow-up outcome data, including early morbidity and mortality, during the first 30 postoperative days, focusing on conduit-associated complications, such as colon conduit ischemia and AL. AL was classified according to the Esophagectomy Complications Consensus Group (ECCG) [7].

Further postoperative diagnostics followed our routine standards. Laboratory parameters were closely monitored, with special focus on inflammatory parameters.

If there were any abnormalities in the postoperative course (e.g., increase in inflammation values, positive blue sample [swallowing test with blue dye on postoperative day 6], clinical deterioration), we conducted additional diagnostics (i.e., CT scan and/or endoscopy). All complications were routinely documented according to the Clavien–Dindo classification (CDC) [17].

### Statistical analysis

Categorical data are expressed as absolute and relative frequencies. Descriptive analyses, including mean and standard deviation, were performed using Microsoft→ Excel 365.

## Results

### Patient characteristics

We documented HSI data for eight patients (80%) from all patients (n = 10) who underwent the described long-segment colon interposition after esophagectomy. In the other two patients (20%), HSI could not be applied because of a technical failure (i.e., the camera was used at the wrong setting, or the image quality was insufficient or out of focus). The patient characteristics are shown in detail in Table 1. The patients’ mean age was 60.4 ± 8.2 years. Mean operative time was 384.6 ± 93.6 min. None of the included patients presented visible atherosclerosis as a comorbidity in preoperative CTA or angiographic visualization. The individual indications for reconstruction with a colon conduit varied in detail among patients (Table 1).

**Table 1:** Patient characteristics including preoperative diagnosis and surgical data, *AL* anastomosis leak, *CTA* computed tomography angiography

Patient’s characteristics	Number of cases (%)
**Sex**	
Male female	6 (75%) 2 (25%)
**Diagnosis**	
Carcinoma Oesophagus Gastric Recessus piriformis Caustic strictures AL after esophagectomy	7 (87,5%) 5 (71%) 1 (14%) 1 (14%) 1 (12,5%) 1 (12,5%)
**Neoadjuvant therapy (only patients with carcinoma)** Cisplatin/Capecitabin (INNOVATION-Study) FLOT (5-Fluorourcil, Leucovorin, Oxaliplatin, Taxan) None	7 1 (14%) 4 (57%) 2 (28%)
**Risk factors**	
abdominal arteriosclerotic disease COPD Diabetes mellitus	0 (0%) 0 (0%) 1 (12,5%)
**Histopatholocial entity (Oesophaguscarcinoma)**	
Squamous cell carcinoma Adenocarcinoma others	0 6 (100%) 0
**Preoperative diagnostic**	
None CTA Angiography	0 (0%) 6 (75%) 2 (25%)
**Surgical technique**	
Left-sided colon interposition Right-sided colon interposition	8 (100%) 0 (0%)
**Operation duration**	
**Operation duration**	
< 360 min > 360 min	3 (37,5%) 5 (62,5%)
**Anastomosis**	
Hand sewn Stapler and overstitching	4 (50%) 4 (50%)
**Procedure**	
One-step-procedure Two-step-procedure	4 (50%) 4 (50%)

### Intraoperative HSI

Intraoperative HSI images were obtained for all the included patients. We defined ROI in the distal region of the conduit (intraabdominally) and the region of the cervical anastomosis for analysis.

**Fig. 5:**
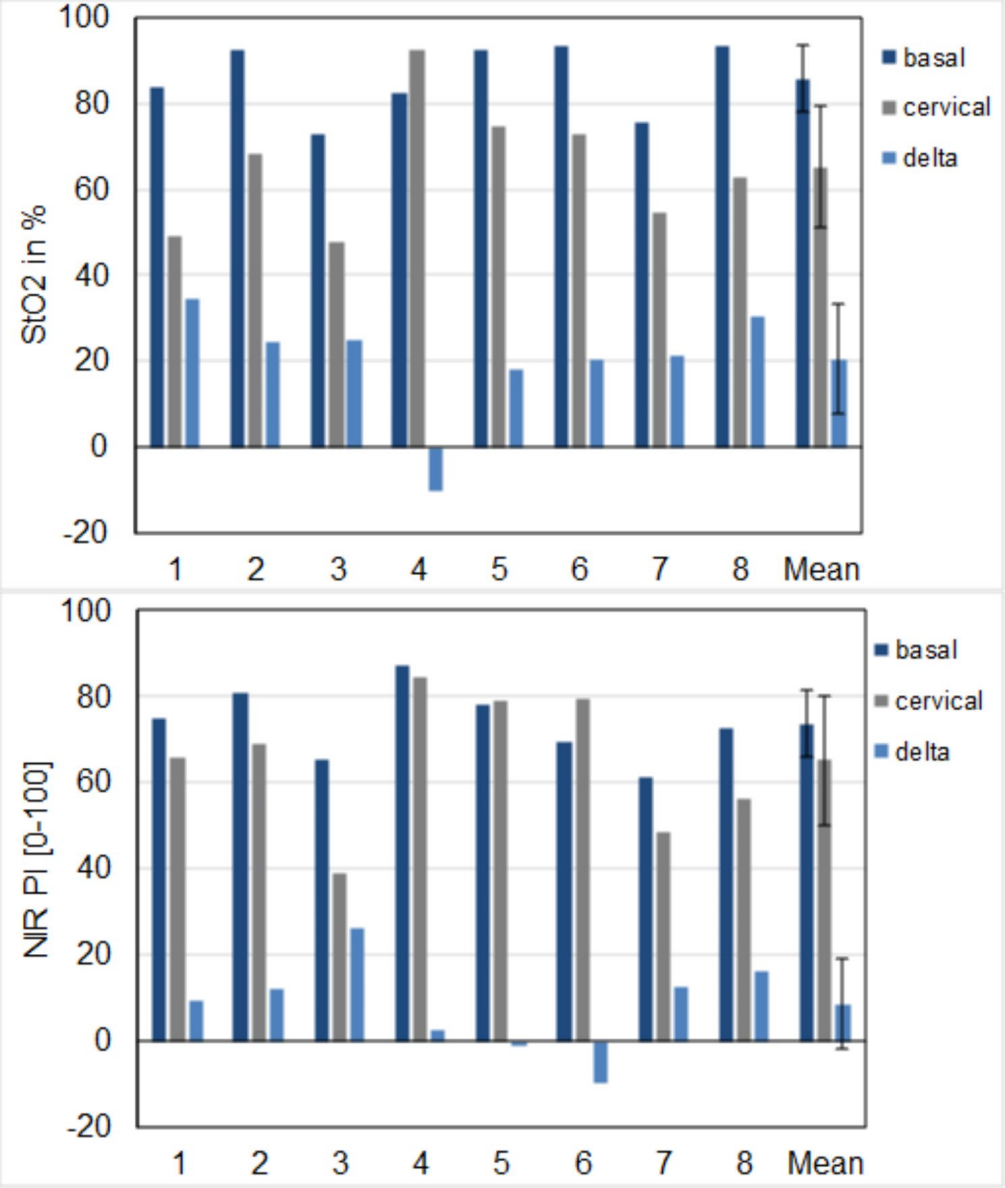
Average values of StO2 and NIR-PI inside the ROI for each patient. The delta bar shows the difference between basal and cervical ROI. The mean and standard deviation across all patients is illustrated on the three right bars

Our evaluation revealed average values of StO_2_ = 85.7 ± 7.9% and NIR-PI = 73.8 ± 7.8 in the distal region of the colon substitute. In addition, the cervical region depiction revealed StO_2_ = 65.2 ± 14.1% and NIR-PI = 65.2 ± 15.0. Detailed data for each patient (1–8) are shown in Fig. 5.

### Postoperative course

The postoperative course and complications according to CDC are shown in detail in Table 2. In summary, no complications occurred in three cases (37.5%).

**Table 2:** postoperative course and complications according to Clavien-Dindo-Classification (CDC)

	Number of cases (%)
**duration of stay**	
≤ 30 days > 30 days > 50 days	5 (62,5%) 3 37,5%) 0 (0%)
**Complications according CDC**	
0 I II IIIa IIIb IV V	3 (37,5%) 0 (0%) 0 (0%) 2 (25%) 2 (25%) 0 (0%) 1 (12,5%)
**90-days-mortality**	0 (%)
**AL**	
No yes	7 (87,5%) 1 (12,5%)

**Fig. 6:**
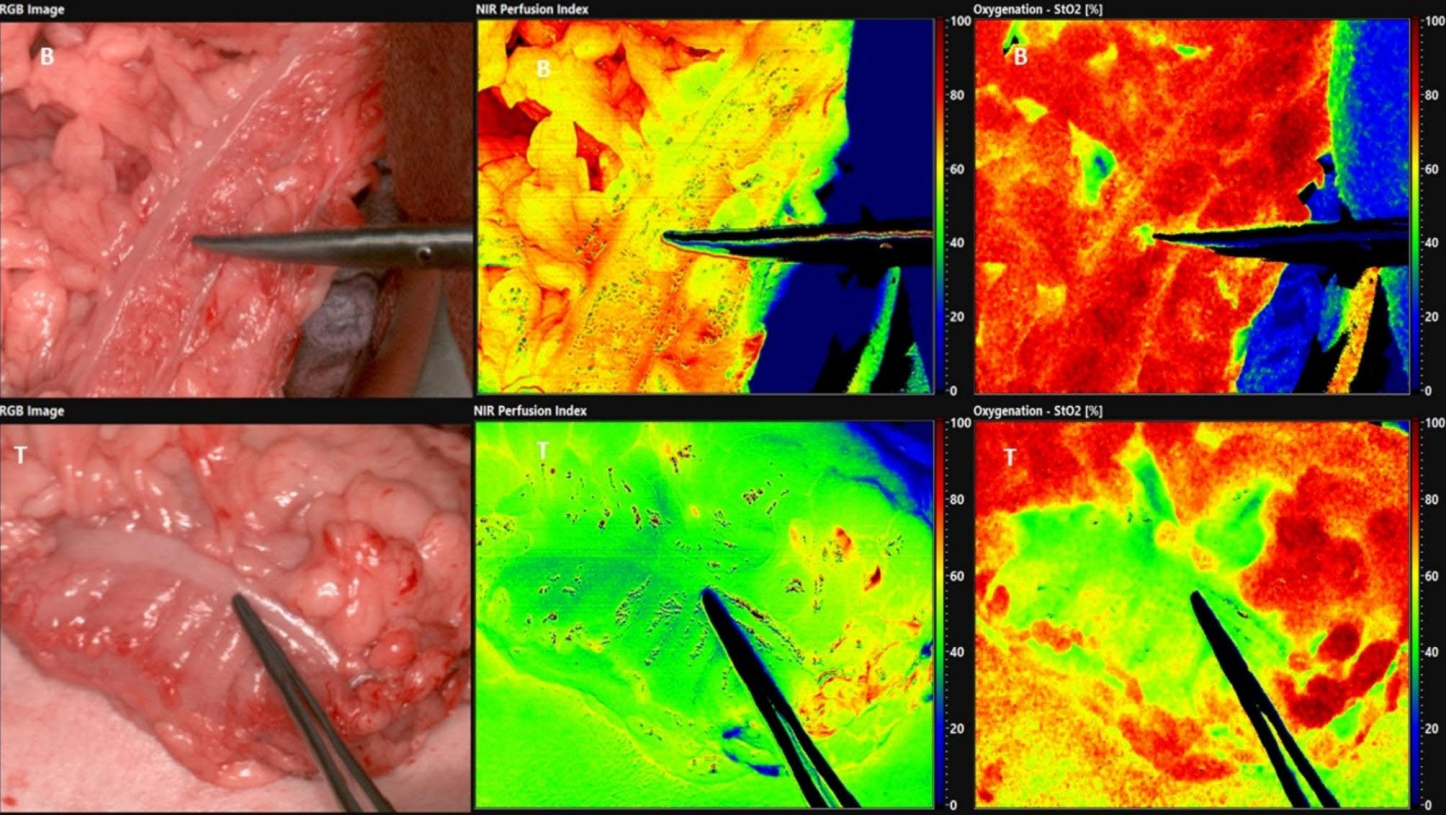
Intraoperative HSI-Measurements of the base (B) and tip (T) of colon interposition in patients with anastomosis leak (AL), *left;* the colored images, *middle;* color-coded images of NIR-PI, *right;* color-coded images of StO2

Grade IIIa complications occurred in two cases (25%). One patient suffered duodenal stump insufficiency, which was drained, and another developed bile leakage after cholecystectomy, which healed by means of a percutaneous bile duct drainage system (PTCD). Two patients (25%) developed grade IIIb complications. In the first case, AL occurred due to necrosis at the tip of the colon conduit, so that the anastomosis had to be surgically reattached. The perfusion parameters of the colon tip were the lowest in this patient (NIR-PI = 39.1, StO_2_ = 47.5%). The difference in NIR-PI between the base and the tip of the colon conduit was also the largest in this patient (Figs. 5 and 6). In the second case, a wound healing disorder occurred, which was closed secondarily via vacuum-assisted closure (VAC). One case (12.5%) had grade V complications. In this case, severe pneumonia with a septic course occurred postoperatively. After prolonged, intensive medical therapy, the patient died due to multi-organ failure on the 53rd postoperative day.

## Discussion

Gastric conduit is the most frequently used esophageal substitute after resection. If the stomach is unsuitable or unavailable as a conduit, a colon segment may be used as the second choice. Colon interposition offers advantages over a gastric conduit, such as its longer length and the remnant esophageal mucosa’s reduced exposure to gastric acid, decreasing the potential for secondary reflux injury.

However, this procedure also has drawbacks. The colon must be free from malignancy or inflammatory bowel disease. Moreover, the operation is complex, as it usually requires a three-cavity incision procedure (abdomen, thorax and neck), at least three anastomoses, and the surgeon must choose (ensuring a sufficient vascular supply) the optimal side for the colonic conduit [1, 18, 19]. Preserving sufficient arterial blood inflow and venous drainage plays an essential intraoperative role. Furthermore, making the right decision when choosing the ideal colon segment (by relying on preoperative and intraoperative data) remains a major challenge for esophageal surgeons [4]. This is why the preoperative assessment of the IMA status via arteriography or CTA when using the left colon and knowing the SMA status in advance (via the same technology) is so important when using the right colon [1, 4, 18–20]. However, McDermott et al. described no significant difference in the rates of ischemic complications following colon interposition in patients who underwent preoperative angiography compared to controls who did not undergo these assessments [21].

Therefore, despite preoperative angiographic findings, an intraoperative assessment of the colon’s arterial blood supply after test clamping all vessels planned for division is important to evaluate the integrity of colon perfusion and exclude major complications, such as postoperative colon ischemia. There are currently three methods to assess the arterial blood flow in the colon conduit intraoperatively, including clamping test, Doppler US, and ICG-FA measurement.

Clamping of the middle colic vessels (left conduit) or ileocolic vessels (right conduit) is a routine test performed at the beginning of surgery and before vessel division. The clinical signs of colon perfusion (serosa-mucosa color, bleeding from the colon’s resection margin) should be checked before the reconstruction with the colon conduit [1]. Pulsation in the marginal artery and ascending left colic artery should be assessed in the mesocolon during surgery [8].

These are subjective tests with no thresholds that may provide us with information to help select the best-perfused colon segment or to determine the need for more investigations. Moreover, the weakness of arterial blood flow in the marginal artery reflects the need to confirm it via another method. The use of intraoperative Doppler US has been demonstrated in other abdominal surgeries [9, 10]; however, the benefit of employing Doppler-US in colon surgery has not been proven [12].

There are no validated US thresholds or well-established algorithms to evaluate arterial blood flow in the marginal artery (Riolan’s arcade) during colorectal surgery that would help surgeons make the best decision intraoperatively regarding which side of the colon to use. Furthermore, none of these tests help surgeons objectively select the best-perfused anastomosis site.

Another approach for evaluating colon perfusion during surgery is ICG-FA. The role of ICG-FA in assessing the anastomosis site has been well established during colorectal surgery [12, 22, 23]. However, this tool still requires dye to be injected, which might have some allergic side effects and contraindications, such hyperthyrosis or severe liver disease [24, 25]. Moreover, ICG-FA is performed using visual assessment of fluorescence angiography (i.e., V-ICG), where the fluorescence signal must be evaluated subjectively. Furthermore, the use of ICG several times before and after dissection of the middle colic vessels might the reduce the confidence of its visual assessment. However, quantifying the ICG-FA (i.e., Q-ICG) intraoperatively is still a novel technology and has been not well established intraoperatively [26].

Consequently, none of these tests provide sufficient information about the perfusion of the colon segment after vascular clamping. Surgeons must select the optimal side of the colon for the conduit and the well-perfused anastomosis site. Furthermore, surgeons must be able to evaluate the need to perform microvascular reconstruction of the middle colic vessels in the case of a poorly perfused colon conduit. Moreover, none of these examination tools (Doppler US, ICG-FA) have been employed in this type of surgery (colon interposition).

In this study, we evaluated the benefit of applying HSI in conjunction with colon interposition with cervical anastomosis. We were able to obtain parameters, such as StO_2_ and NIR-PI, of the colon conduit after a vascular clamping test. We observed no obvious differences between parameters at the colon conduit’s base and its tip (< 20%) in most of our patients. Furthermore, we identified only one patient (No. 3) with AL type III according to the ischemia at the anastomosis site. The StO_2_ and NIR-PI values at the tip of the colon conduit (anastomosis site) were reduced (StO_2_ = 47.5%, NIR-PI = 39.1) in this patient (Fig. 5). This case had the largest difference in NIR-PI values (Δ = 26.4) between the tip and base of the colon conduit (Fig. 5). However, as his anastomosis site exhibited normal clinical signs of good perfusion, such as bleeding and the serosa-mucosa color, we performed the anastomosis by relying on our subjective assessment. This information was apparently inadequate, and he required a re-anastomosis. We detected no ischemia in the colon conduit in all included patients, nor was there any need to change the side of the colon interposition graft (i.e., from left to right). However, HSI- measurements showed poor perfusion of the subjectively marked anastomosis site and should have been adapted to the proximal in line with the HSI data. Furthermore, applied intraoperatively, HSI has already been proven as a sufficient tool in the assessment of tissue perfusion in many surgical disciplines, including colorectal, esophageal, and pancreatic surgery.[14, 16, 27, 28]. To the best of our knowledge, HSI has not been used in this type of abdominal surgery before. We managed to acquire perfusion parameters from the colon conduit, which helped us to select the side of colon interposition and anastomosis site intraoperatively. Furthermore, our patients’ AL rates after this image-guided surgical procedure were similar to those in other published studies [29–31].

HSI is a valuable and non-invasive, contact-free method to objectively assess colon conduit perfusion.

The limitation of the current study was the small number of included patients and the absence of a control group. However, this was a single-center study that involved a seldom-complex operation; therefore, a further multicenter study is needed. Nonetheless, this study represents a new method for detecting the perfusion of a colon conduit in an uncommon operation.

## Conclusion

Our most recent data indicate that employing HSI to assess colon-conduit perfusion helps to prevent ischemic complications and reduce postoperative mortality. HSI can be applied safely and easily by relying on standard measurement points when employing a colon conduit for reconstruction after esophagectomy/esophagogastrectomy.

## Data Availability

The datasets used and/or analysed during the current study available from the corresponding author on reasonable request.
